# Conventional versus Hepatic Arteriography and C-Arm CT-Guided Ablation of Liver Tumors (HepACAGA): A Comparative Analysis

**DOI:** 10.3390/cancers16101925

**Published:** 2024-05-18

**Authors:** Niek Wijnen, Rutger C. G. Bruijnen, Evert-Jan P. A. Vonken, Hugo W. A. M. de Jong, Joep de Bruijne, Guus M. Bol, Jeroen Hagendoorn, Martijn P. W. Intven, Maarten L. J. Smits

**Affiliations:** 1 Department of Radiology and Nuclear Medicine, University Medical Center Utrecht, 3584 CX Utrecht, The Netherlands; 2 Department of Gastroenterology and Hepatology, University Medical Center Utrecht, 3584 CX Utrecht, The Netherlands; 3 Department of Medical Oncology, University Medical Center Utrecht, 3584 CX Utrecht, The Netherlands; 4 Department of Radiotherapy, University Medical Center Utrecht, 3584 CX Utrecht, The Netherlands; 5 Department of Surgery, University Medical Center Utrecht, 3584 CX Utrecht, The Netherlands

**Keywords:** C-arm CT, colorectal liver metastases, CTHA, hepatocellular carcinoma, local tumor recurrence, microwave ablation, radiofrequency ablation

## Abstract

**Simple Summary:**

Catheter-assisted ablations have shown significant improvements in the outcomes of percutaneous thermal ablation for liver cancer. A novel approach within this category is the Hepatic Arteriography and C-Arm CT-Guided Ablation (HepACAGA) technique, which integrates C-arm CT hepatic arteriography with C-arm guided navigation. This cohort study aimed to assess the effectiveness and safety of the HepACAGA technique compared to conventional ultrasound/CT-guided thermal ablation in treating hepatocellular carcinoma and colorectal liver metastasis. A total of 68 patients with 120 tumors undergoing HepACAGA and 53 patients with 78 tumors undergoing conventional ablation were included. The HepACAGA technique demonstrated superior outcomes: lower rates of local tumor recurrence, longer local tumor recurrence-free survival, and fewer procedure-related complications. These findings suggest that HepACAGA is a safer and more effective ablation technique in liver cancer treatment compared to conventional ablation methods.

**Abstract:**

Purpose: Hepatic Arteriography and C-Arm CT-Guided Ablation of liver tumors (HepACAGA) is a novel technique, combining hepatic–arterial contrast injection with C-arm CT-guided navigation. This study compared the outcomes of the HepACAGA technique with patients treated with conventional ultrasound (US) and/or CT-guided ablation. Materials and Methods: In this retrospective cohort study, all consecutive patients with hepatocellular carcinoma (HCC) or colorectal liver metastases (CRLM) treated with conventional US-/CT-guided ablation between 1 January 2015, and 31 December 2020, and patients treated with HepACAGA between 1 January 2021, and 31 October 2023, were included. The primary outcome was local tumor recurrence-free survival (LTRFS). Secondary outcomes included the local tumor recurrence (LTR) rate and complication rate. Results: 68 patients (120 tumors) were included in the HepACAGA cohort and 53 patients (78 tumors) were included in the conventional cohort. In both cohorts, HCC was the predominant tumor type (63% and 73%, respectively). In the HepACAGA cohort, all patients received microwave ablation. Radiofrequency ablation was the main ablation technique in the conventional group (78%). LTRFS was significantly longer for patients treated with the HepACAGA technique (*p* = 0.015). Both LTR and the complication rate were significantly lower in the HepACAGA cohort compared to the conventional cohort (LTR 5% vs. 26%, respectively; *p* < 0.001) (complication rate 4% vs. 15%, respectively; *p* = 0.041). Conclusions: In this study, the HepACAGA technique was safer and more effective than conventional ablation for HCC and CRLM, resulting in lower rates of local tumor recurrence, longer local tumor recurrence-free survival and fewer procedure-related complications.

## 1. Introduction

Primary and secondary hepatic malignancies are frequently encountered in clinical practice. Hepatocellular carcinoma (HCC), the most prevalent form of primary liver cancer, is ranked as the third leading cause of cancer-related mortality worldwide; and colorectal cancer (CRC) predominates as the primary cancer type giving rise to metastatic liver cancer [1,2]. At least 25–50% of CRC patients will develop colorectal liver metastases (CRLM) during the course of their disease [3]. Currently, surgical resection is the gold standard for curative treatment of hepatic malignancies. However, only 10–20% of the patients with liver cancer are considered suitable candidates for surgical resection at the time of diagnosis [4].

Thermal ablation is an established alternative local treatment option for hepatocellular carcinoma and liver metastases. Due to the minimally invasive nature of image-guided thermal ablation and outcomes comparable to surgical resection, it has become a treatment of choice in several guidelines [5,6].

Thermal ablation is, however, not a singular, uniform treatment option. Techniques and outcomes of thermal ablation vary widely [7,8]. There are major differences in ablation technologies used, such as radiofrequency ablation (RFA), microwave ablation (MWA), and cryoablation. Even within these categories, large differences exist between products and imaging techniques.

Several image-guidance techniques are available to facilitate the needle placement in the target. Ultrasound (US) is frequently used for ablation guidance. However, many liver tumors are occult on US and one cannot reliably monitor the ablation zone with US [9,10]. CT is also frequently used for guiding thermal ablation procedures. A downside of CT is that most liver tumors can only be distinguished with (a substantial dose of) intravenous contrast agent and many tumors are occult even on contrast-enhanced CT [9,11].

CT hepatic arteriography (CTHA) is an important development that has greatly improved the outcomes of liver ablation [12,13,14]. CTHA is obtained by inserting a catheter into the hepatic artery (via femoral or radial access) and performing CT during intra-arterial contrast injection. Compared to intravenous contrast injection, selective intra-arterial contrast administration significantly improves tumor visualization. A drawback of CTHA is the need for patient transfer between the angiography room (for catheter placement) and the CT room (for CT-guided puncture) for institutions without a hybrid angiography–CT room. Patient transfer increases the risk of catheter dislodgment and contamination. Moreover, this two-room procedure can pose logistical challenges [15].

The HepACAGA technique (Hepatic Arteriography and C-Arm CT-Guided Ablation) is a combination of C-arm CTHA and C-arm guided navigation. This combination allows for the entire procedure to be conducted in the angiography suite, obviating the need for patient transfer. Also, C-arm guidance software greatly facilitates angulated needle placement and embolization can be readily performed in the case of post-ablation hemorrhage or if combined embolization and ablation were to be desired.

The initial results of HepACAGA were promising, with a high technical success rate and a low tumor recurrence rate [15]. The aim of this study was to compare the outcomes of patients treated with HepACAGA with patients treated with conventional US-/CT-guided ablation.

## 2. Materials and Methods

### 2.1. Ethical Approval

The data used in this study were extracted from the ‘Minimally Invasive Thermal Ablation (MITA) study’ database, which is a prospective registry of patients undergoing thermal ablation of liver tumors at the University Medical Center Utrecht (Utrecht, The Netherlands). Permission for the MITA database was granted by the local ethical institutional review board (No. 21/709). All included patients provided written informed consent.

### 2.2. Patients

In this retrospective cohort study, all consecutive patients with hepatocellular carcinoma (HCC) or colorectal liver metastases (CRLM) treated with conventional ablation between 1 January 2015, and 31 December 2020 (conventional cohort), and patients treated with HepACAGA between 1 January 2021, and 31 October 2023 (HepACAGA cohort) were included. 1 January 2021 marks the moment that the HepACAGA technique became the primary treatment method at our institute.

Patients were excluded under the following circumstances: 1. no imaging follow-up; 2. peroperative ablations; 3. re-ablations of lesions with local recurrence after prior local treatment; 4. ablation combined with another local treatment; and 5. microwave ablations (MWA) performed with an MWA ECO system.

### 2.3. Treatment

Flowcharts outlining the procedural steps of the HepACAGA technique and conventional ablation method are provided in the Supplementary Materials (Figure S1).

#### 2.3.1. Conventional Procedure

Conventional percutaneous thermal ablation procedures were performed under general anesthesia and were guided by US and/or CT imaging in the CT room. Ablation needles were placed under real-time US or CT guidance. For CT guidance, iodine contrast agent (Ultravist, Bayer, Leverkusen, Germany) was administered intravenously. Upon confirmation of the correct position of the needle, ablation was initiated. Ablations were carried out using RFA, MWA or cryoablation. Ablation margins were assessed using contrast-enhanced CT. If the margin was deemed inadequate, the needle was repositioned for additional ablation. When adequate, the needle was retracted under tract ablation.

#### 2.3.2. HepACAGA Procedure

The entire HepACAGA procedure was conducted in the angiography suite, as described previously [15]. In short, under general anesthesia the common femoral artery was punctured and a catheter advanced into the hepatic artery. C-arm CTHA was acquired under apnea with the intra-arterial pump injection of small volumes of contrast agent (Visipaque, GE HealthCare, Chicago, IL, USA) (2:1 dilution with NaCl, flow rate 0.5–2.0 mL/s, total amount 10–40 mL). The target lesion was selected on CTHA and a needle trajectory was planned using the C-arm navigation software (XperGuide, Philips, Best, The Netherlands, https://www.usa.philips.com/healthcare/product/HCOPT06/xperguide-live-3d-needle-guidance (accessed on 7 May 2024)). Next, the antenna was introduced in accordance with the planned trajectory under real-time fluoroscopy guidance. All HepACAGA procedures were conducted with MWA (Emprint^®^ HP, Medtronic, Dublin, Ireland). At each needle placement, apnea was induced by temporarily pausing the ventilator, resulting in automatic lung deflation, mimicking the position of the liver as observed during the planning C-arm CT. Thermal ablation was initiated once C-arm CTHA confirmed a correct needle position. C-arm CTHA was repeated immediately after the ablation. Pre- and post-ablation C-arm CTs were semi-automatically fused (using XperGuide software) to assess adequate ablation margins. Additional ablation was performed if ablation margins were deemed inadequate. Finally, tract ablation was carried out upon retraction of the antenna.

### 2.4. Imaging Follow-Up

Imaging follow-up consisted of an initial MRI or CT scan 1 month after the thermal ablation to assess treatment efficacy, complications, and local tumor recurrence. Thereafter, patients underwent MRI or CT scans every 3–6 months.

### 2.5. Study Objectives

The consensus guidelines for the definition of time-to-event end points in image-guided tumor ablation were used for the reported outcomes [16].

#### 2.5.1. Local Tumor Recurrence

The primary objective of our study was to compare the local tumor recurrence-free survival (LTRFS), including the 1-year and 2-year LTRFS rates, following percutaneous thermal ablation performed using the HepACAGA technique with conventional ablation. Radiology reports from follow-up imaging were used to assess the treatment effect and to determine if there was local tumor recurrence (LTR) following the percutaneous thermal ablation. Any indication of residual or local recurrence of the tumor at the site of the ablation zone was considered as LTR. For the per-lesion analysis, LTRFS was defined as the duration from the date of ablation until local recurrence of the tumor was observed. Non-recurrent tumors were censored at the last follow-up assessment. For the per-patient analysis, LTRFS was defined as the duration from the date of ablation until local recurrence of any ablated tumor was observed. Patients were censored at the last follow-up assessment.

LTR rates were also compared between the two techniques, defined as the percentage of recurrent tumor at the ablation site during follow-up. Subanalyses were conducted to evaluate LTR rates separately for each tumor type and for ablations performed with MWA.

Overall survival (OS) was defined as the duration from the date of ablation until the event of death by any cause.

#### 2.5.2. Technical Success and Procedure-Related Characteristics

Technical success was defined as the successful completion of a percutaneous ablation procedure with the planned technique (either HepACAGA or conventional ablation). In-room time and procedure duration were retrieved from the anesthesiology reports. In-room time was specified as the interval between patient arrival and departure, while procedure duration was defined as the interval between the start and end of the procedure. A per-lesion procedure time was computed by dividing the total procedure time by the number of treated lesions.

In addition, the amount of administered contrast agent and the radiation dose received by the patient were retrieved from the anesthesiology and radiology reports, respectively. The C-arm CT utilized in the HepACAGA technique quantified the radiation dose as the total DAP (dose area product, Gy cm^2^), whereas the CT scanner used in conventional ablation measured it as the total DLP (dose length product, mGy cm). To enable a comparison of the radiation dose between both techniques, the total DAP and total DLP measurements were converted to effective dose (mSv) by multiplying them with conversion factors. For the total DAP and total DLP, a conversion factor of 0.38 mSv Gy^−1^ cm^−2^ and 0.015 mSv mGy^−1^ cm^−1^ was used, respectively. These conversion factors were specified for the used (C-arm) CT system [17,18]. Also, a per-lesion analysis was conducted for administered contrast and effective dose.

The technical success and procedure-related characteristics were compared between ablations performed with the HepACAGA technique and the conventional ablation method.

#### 2.5.3. Complications

To evaluate safety differences between the HepACAGA technique and conventional ablations, patient records were examined for intraprocedural or postprocedural complications associated with the ablation. Complications were graded according to the Common Terminology Criteria for Adverse Events (CTCAE), version 5 [19]. Perioperative mortality, defined as death occurring within 30 days after ablation, was also assessed separately.

### 2.6. Statistical Analysis

Differences in baseline characteristics between the patients included in the HepACAGA cohort and the conventional cohort were analyzed using unpaired *t*-tests to compare continuous variables, and Pearson Chi-Square (Χ^2^) tests for categorical variables.

LTRFS and OS were estimated with Kaplan–Meier survival curves and compared using the log-rank test. Corresponding hazard ratios (HR) were obtained with Cox proportional hazards regression analysis.

The LTR rates, technical success, and complication rate were compared between the HepACAGA cohort and conventional cohort using Fisher’s exact tests. Procedure-related time parameters, contrast administration and the effective dose were assessed by comparing means using unpaired *t*-tests.

Statistical analyses were carried out using the R software package, version 4.3.2 (R Foundation, Vienna, Austria) [20]. *p*-values below 0.05 were considered statistically significant.

## 3. Results

### 3.1. Patient Inclusion and Characteristics

**Figure 1:**
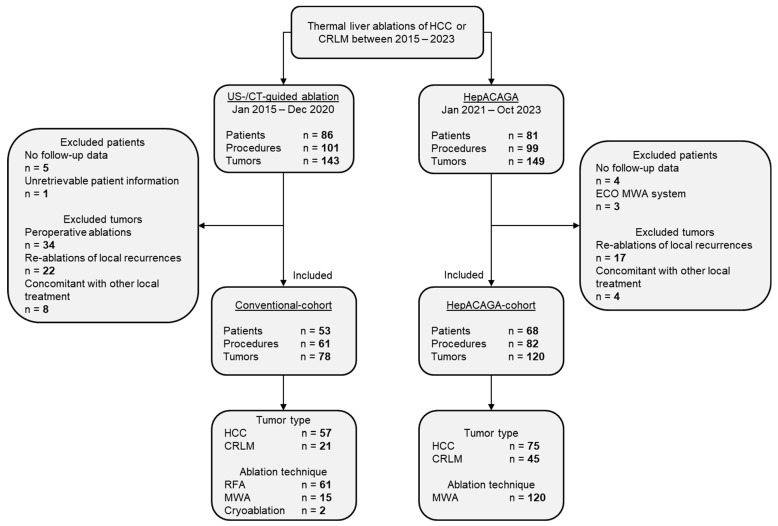
Flowchart demonstrating patient selection.

In the study periods, a total of 86 patients diagnosed with HCC or CRLM (143 tumors) underwent conventional ablations and 81 patients (149 tumors) were treated with the HepACAGA technique. After the exclusion criteria were applied, 53 patients (78 tumors) were included in the conventional cohort, and 68 patients (120 tumors) were included in the HepACAGA cohort (Figure 1).

Table 1 presents the baseline characteristics for each cohort. Age, gender and BMI were comparable between the HepACAGA and conventional cohort. HCC was the predominant tumor type, accounting for 75/120 tumors (63%) in the HepACAGA cohort and 57/78 tumors (73%) in the conventional cohort. While all HepACAGA procedures were performed with MWA, the primary ablation modality for conventional ablations was RFA (61/78, 78%), followed by MWA (15/78, 19%) and cryoablation (2/78, 3%). Although the median tumor size was equal between cohorts (15 mm), the HepACAGA cohort contained both smaller (1–10 mm) and larger lesions (>31 mm) compared to conventional ablations.

### 3.2. Local Tumor Recurrence

The median imaging follow-up for the HepACAGA cohort was 12 months (range 1–34 months), and for the conventional cohort 20 months (range 1–72 months) (Table 2).

A recurrent tumor was identified at the ablation site during follow-up in 6/119 tumors (5%) in the HepACAGA cohort versus 20/76 tumors (26%) in the conventional cohort (*p* < 0.001). In the per-patient analysis, the LTR rate was 4/67 patients (6%) in the HepACAGA cohort versus 14/51 patients (27%) in the conventional cohort (*p* = 0.002). The outcomes of the subanalyses for each tumor type and ablations performed with MWA are presented in Table 2.

**Figure 2:**
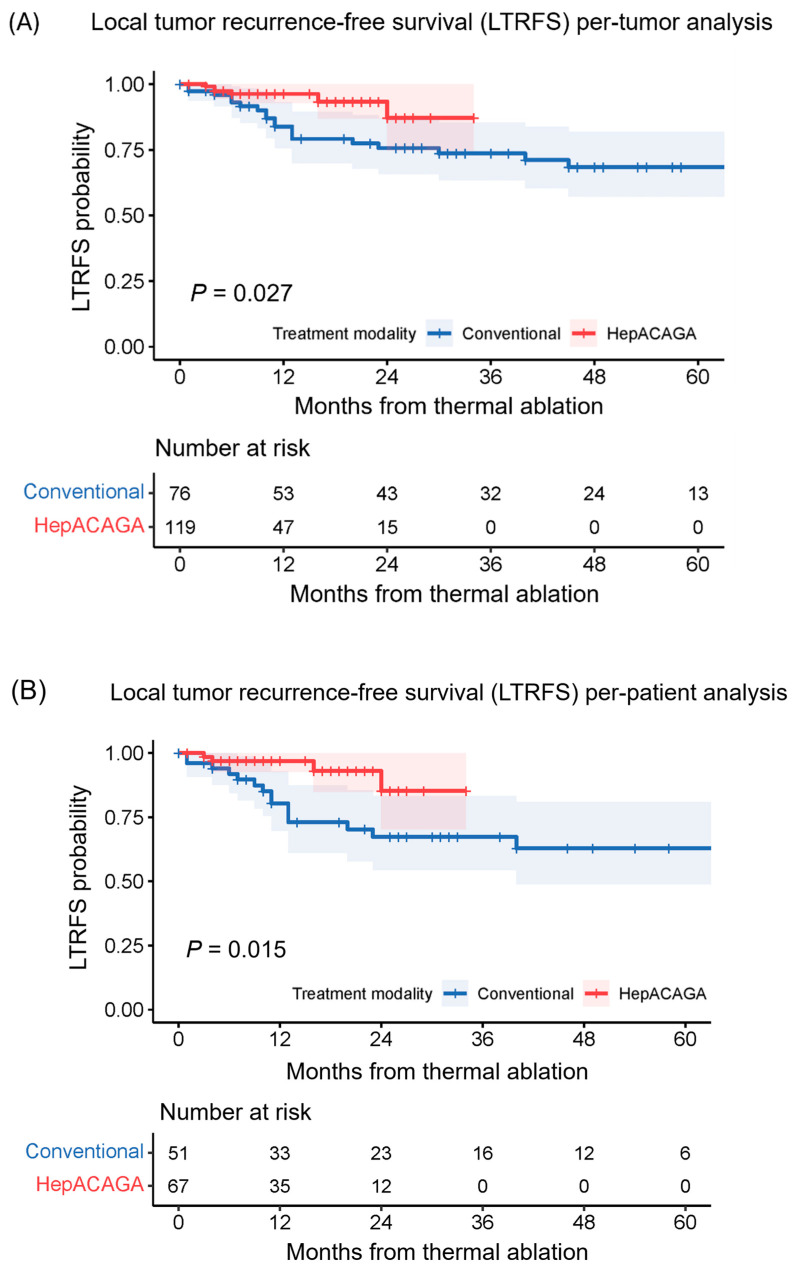
Kaplan–Meier survival curves illustrating the local tumor recurrence-free survival (LTRFS) with 95% CI for both techniques. Log-rank tests were used for comparison. The number at risk corresponds to either the number of tumors or the number of patients present at each time point. (**A**) represents the per-tumor analysis; (**B**) demonstrates the per-patient analysis.

LTRFS was significantly longer in the HepACAGA cohort (median LTRFS not reached) versus the conventional cohort (median LTRFS = 71 months) (HR: 0.274 [95% CI: 0.089–0.838]; *p* = 0.015) (Figure 2). The per-patient 1-year LTRFS (97% vs. 80%, *p* = 0.002) and 2-year LTRFS (85% vs. 67%, *p* = 0.009) were also significantly higher for patients treated with the HepACAGA technique.

### 3.3. Overall Survival

**Figure 3:**
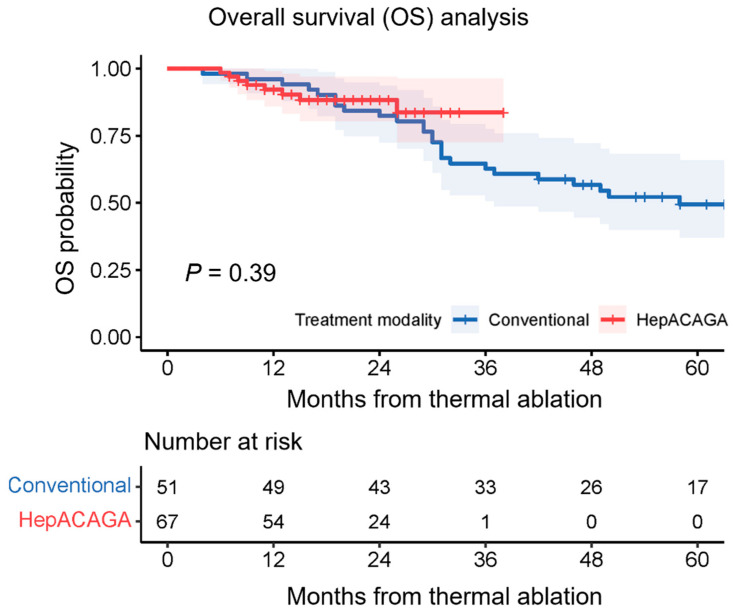
Kaplan–Meier curves illustrating the overall survival (OS) with 95% CI for each treatment modality. A log-rank test was conducted for comparative analysis. The number at risk corresponds to number of patients present at each time point.

During follow-up, 8/67 patients (12%) of the HepACAGA cohort died versus 26/51 patients (51%) of the conventional cohort. Overall survival was not significantly different between cohorts (HR: 0.695 [95% CI: 0.292–1.656]; *p* = 0.39) (Figure 3). Overall survival is separately presented for each tumor type in the Supplementary Materials (Figure S2).

### 3.4. Technical Success

With HepACAGA, 81/82 of ablations were technically successful (99%) (Table 3). One procedure failed due to the inability to separate the stomach from the liver using both hydrodissection and pneumodissection. Conventional ablation was successfully performed in 59/61 procedures (97%). One procedure was terminated due to inaccurate transpulmonary needle-placement, which resulted in a pneumothorax. Another procedure was discontinued because the lesion was not visible with US.

### 3.5. Procedure-Related Characteristics

Procedure times were longer for the HepACAGA technique (Table 3). Specifically, the median per-lesion procedure time for the HepACAGA technique was 59 min (range 16–137) versus 45 min (range 24–119) for conventional ablations. The median amount of contrast agent used per ablated lesion was lower for HepACAGA procedures than for conventional ablation (100 mL vs. 140 mL, respectively), whereas the median effective dose (per ablated lesion) was higher (39 mSv vs. 11 mSv, respectively).

### 3.6. Complications

The complication rate (grade 2 and higher) was significantly lower in the HepACAGA cohort (3/82 procedures, 4%) than in the conventional cohort (9/61 procedures, 15%) (*p* = 0.041) (Table 3). Notably, pneumothorax was the most common complication in conventional ablations (5/61, 8%), whereas none occurred with HepACAGA. Both ablation techniques resulted in one Grade 4 (CTCAE v5) complication, which was an active bleeding requiring embolization. There were no procedure-related mortalities in either cohort (defined as death occurring within 30 days after ablation).

## 4. Discussion

We showed that patients treated with the HepACAGA technique had a lower per-tumor local tumor recurrence rate (5% vs. 26%), longer local tumor recurrence-free survival (HR: 0.274, *p* = 0.015), and a lower complication rate (4% vs. 15%) compared with patients treated using conventional ablation methods.

Although there is a wide variation in techniques for the thermal ablation of liver tumors, most of these contain the following four components: 1. imaging techniques to localize the target and non-targets (e.g., CT, US, MRI, PET, and image fusion); 2. navigation techniques to guide needle placement (e.g., free-hand, needle guide, stereotactics, robotics); 3. ablation modality to obtain the ablative effect (e.g., needle, generator); and 4. margin assessment techniques to verify adequate ablation margins all around the tumor (e.g., eye-balling, manual or semi-automatic fusion, and automatic assessment). The HepACAGA technique differs from the conventional technique in three of these four components: 1. it uses C-arm CTHA instead of US or CT with intravenous contrast injection; 2. it relies on C-arm navigation (XperGuide) instead of free-hand or US needle guidance; and 4. margin assessment is performed with semi-automatic fusion instead of eye-balling. All these factors may have contributed to the superior outcomes of the HepACAGA technique in this study.

LTRFS (including 1-year and 2-year LTRFS) rates were significantly higher in the HepACAGA cohort and LTR was reduced by 81%. These results are also supported by interim subgroup analysis of the COLLISION trial, where CRLM patients treated with catheter-assisted ablation exhibited a statistically significantly better LTRFS and lower LTR compared to patients treated with conventional ablation [21,22].

The complication rate was reduced by 73% in the HepACAGA cohort compared to conventional ablations. Both techniques resulted in one case of active post-ablation bleeding that required embolization. The advantage with HepACAGA was that embolization could be performed without any delay since the patient was already in the angiography suite. Pneumothorax was the most common complication overall, occurring in 8% of the conventional ablations, while none were observed in the HepACAGA procedures (Table 3). The absence of pneumothoraces in the HepACAGA cohort was likely due to the increased freedom in angulation when puncturing under C-arm guidance. C-arm guidance facilitates needle trajectories out of the axial plane with steep caudo-cranial angulation, enabling access to lesions located high in the liver dome without traversing the lungs, as opposed to conventional ablations requiring a transpulmonary route in the axial plane.

There are several limitations of the HepACAGA technique. A general limitation of CTHA is that it involves catheterization, which poses a small risk of puncture site bleeding or infection. However, none of the patients in the HepACAGA cohort of this study experienced such complications. Another drawback of HepACAGA is that it requires general anesthesia. This is necessary to induce apneas during C-arm CTs and needle placement. Also, the needle is placed under real-time fluoroscopy, resulting in additional radiation exposure. The median per-lesion effective dose for HepACAGA was higher (+28 mSv) compared to the conventional cohort. This can be attributed to the fact that the majority (69%) of conventional ablations were performed using US guidance, not involving radiation. Also, the median per-lesion procedure time for HepACAGA was slightly longer (+14 min) compared to the conventional ablation. The median contrast administration was lower for HepACAGA (−40 mL per lesion). We consider the additional procedure time and additional radiation exposure a minor trade-off for the significantly improved outcomes achieved with the HepACAGA technique.

Limitations of this study also need to be addressed. First, all HepACAGA procedures were performed with MWA, whereas RFA was the primary modality in the conventional cohort. Our practice has gradually shifted from using RFA as the primary ablation modality to using MWA because of advantages such as larger, more spherical, single-needle ablation zones, a shorter ablation time, reduced susceptibility to the heat-sink effect, and the ability to control the size and shape of the ablation zone [23,24]. The application of MWA in all HepACAGA procedures might have contributed to the favorable outcomes observed in the HepACAGA cohort. However, subanalysis of ablations performed with MWA demonstrated a comparable difference in outcomes, with significantly improved LTR in the HepACAGA cohort compared to conventional ablations (5% vs. 33%). This indicates that the impact of the bias in ablation modality is probably limited (Table 2).

Second, the median follow-up period for the HepACAGA cohort (12 months; range 1–34 months) was inevitably shorter than the conventional (historical) cohort. Consequently, some patients in the HepACAGA cohort may still develop LTR. However, the majority of LTRs are detected within the first nine months after treatment [25]. Additionally, a higher percentage of HepACAGA patients underwent MRI (90% vs. 96%) for follow-up, probably enabling the earlier detection of local recurrences.

Other limitations are the retrospective design, limited number of patients and difference in tumor characteristics between cohorts. A notable difference in tumor characteristics is the higher number of both small lesions (1–10 mm diameter) and large lesions (>31 mm) in the HepACAGA cohort. A potential explanation for this difference is the increased confidence in being able to detect and successfully ablate both small- and large-sized lesions with the HepACAGA technique. Small lesions can be hard to ablate due to difficulty in detecting the lesion, and large lesions can be hard to ablate with a sufficient margin [25,26]. At our institute, the HepACAGA technique is now used for all patients referred for liver ablation (including liver metastases from a neuroendocrine tumor, melanoma, and breast cancer). To minimize heterogeneity within the study population, we only included HCC and CRLM patients in this study.

The HepACAGA technique represents one among several variant approaches to perform liver tumor ablation with intra-arterial contrast injection. In essence, CTHA facilitates the identification of the target lesions and assessment of the ablation zone. The implementation of CTHA for liver ablations is gaining ground, with several international centers currently adopting this technique as a routine practice [27]. If the long-term outcomes of CTHA-assisted ablation prove superior to conventional ablation techniques, it has the potential to become the standard approach for liver tumor ablations worldwide.

## 5. Conclusions

In conclusion, the HepACAGA technique had superior outcomes compared to a historic cohort of conventional ablation for HCC and CRLM combined, resulting in lower rates of local tumor recurrence, longer local tumor recurrence-free survival and fewer procedure-related complications.

## Figures and Tables

**Table 1:** Baseline characteristics.

		Conventional Ablation	HepACAGA	*p* Value
*Patient-related characteristics*		*n* = 53	*n* = 68	
Sex, *n* (%)	Male	36 (68)	51 (75)	0.390 *
	Female	17 (32)	17 (25)	
Age (years), median (range)		64 (38–86)	68 (16–87)	0.102 ^†^
BMI (kg/m^2^), median (range)		25 (17–34)	27 (16–42)	0.301 ^†^
ASA, *n* (%)	1	1 (2)	4 (6)	0.270 *
	2	27 (51)	22 (32)	
	3	21 (40)	35 (51)	
	4	1 (2)	3 (4)	
	Unknown	3 (6)	4 (6)	
*Tumor-related characteristics*		*n* = 78	*n* = 120	
Tumor type, *n* (%)	HCC	57 (73)	75 (63)	0.123 *
	CRLM	21 (27)	45 (37)	
Diameter (mm), median (range)		15 (5–32)	15 (2–46)	0.303 ^†^
Size (mm), *n* (%)	1–10	14 (18)	33 (28)	0.021 *
	11–20	41 (53)	58 (48)	
	21–30	22 (28)	19 (16)	
	>31	1 (1)	10 (8)	
*Procedure-related characteristics*		*n* = 78	*n* = 120	
Image guidance for needle placement, *n* (%)	Percutaneous US	54 (69)	–	
	CT fluoroscopy	24 (31)	–	
	C-arm CT	–	120 (100)	
Ablation modality, *n* (%)	RFA	61 (78)	–	
	MWA	15 (19)	120 (100)	
	Cryoablation	2 (3)	–	

ASA = American Society of Anaesthesiologists; BMI = Body Mass Index; CRLM = Colorectal liver metastasis; HCC = Hepatocellular carcinoma; RFA = Radiofrequency ablation; MWA = Microwave ablation. * Chi-Square (χ^2^) test. ^†^ Unpaired *t*-test.

**Table 2:** Follow-up.

		Conventional Ablation	HepACAGA	*p* Value
*Follow-up analysis*		*n* = 51	*n* = 67	
Follow-up (months), median (range)		20 (1–72)	12 (1–34)	<0.001 ^†^
Follow-up imaging modality, *n* (%)	MRI only	21 (41)	39 (58)	0.184 *
	CT only	5 (10)	2 (3)	
	MRI + CT	21 (41)	23 (34)	
	MRI + ^18F^FDG-PET	4 (8)	3 (4)	
New liver tumors (other than ablated), *n* (%)		19 (37)	20 (30)	0.397 *
New or progressive extrahepatic tumors, *n* (%)		13 (25)	14 (21)	0.556 *
LTR per patient, *n* (%)		14 (27)	4 (6)	0.002 ^‡^
		*n* = 76	*n* = 119	
LTR per tumor, *n* (%)		20 (26)	6 (5)	<0.001 ^‡^
*LTR subanalysis HCC*		*n* = 33	*n* = 38	
LTR per patient, *n* (%)		9 (22)	2 (5)	0.019 ^‡^
		*n* = 55	*n* = 74	
LTR per tumor, *n* (%)		14 (25)	2 (3)	<0.001 ^‡^
*LTR subanalysis CRLM*		*n* = 18	*n* = 29	
LTR per patient, *n* (%)		5 (27)	2 (7)	0.089 ^‡^
		*n* = 21	*n* = 45	
LTR per tumor, *n* (%)		6 (29)	4 (8)	0.062 ^‡^
*LTR subanalysis MWA*		*n* = 12	*n* = 67	
LTR per patient, *n* (%)		4 (33)	4 (6)	0.016 ^‡^
		*n* = 15	*n* = 119	
LTR per tumor, *n* (%)		5 (33)	6 (5)	0.003 ^‡^

CRLM = Colorectal liver metastasis; HCC = Hepatocellular carcinoma; LTR = Local tumor recurrence; MWA = Microwave ablation. In comparison to the baseline table there were less patients and tumors in both cohorts, attributable to unsuccessful ablation, and therefore no follow-up data. * Chi-Square (χ^2^) test. ^†^ Unpaired *t*-test. ^‡^ Fisher’s exact test.

**Table 3:** Procedure-related outcomes.

		Conventional Ablation	HepACAGA	*p* Value
*Technical Success*		*n* = 61	*n* = 82	
Technically successful procedures, *n* (%)		59 (97)	81 (99)	0.575 ^‡^
*Complications*				
Complicated procedures, *n* (%)		9 (15)	3 (4)	0.041 ^‡^
Complications (CTCAE v5), *n* (%)	Grade 2	3 (5)	1 (1)	0.641 *
	Grade 3	5 (8)	1 (1)	
	Grade 4	1 (2)	1 (1)	
Type of complication, *n* (%)	Pneumothorax	5 (8)	–	
	Perihepatic hematoma	2 (3)	1 (1)	
	Subcapsular hematoma	1 (2)	–	
	Active bleeding (embolization required)	1 (2)	1 (1)	
	Abscess	1 (2)	1 (1)	
*Time parameters*				
In-room time (min), median (range)		106 (61–162)	135 (73–236)	<0.001 ^†^
Procedure duration (min), median (range)		60 (24–119)	86 (43–179)	<0.001 ^†^
Per-lesion procedure time (min), median (range)		45 (24–119)	59 (16–137)	<0.001 ^†^
*Contrast and effective dose*				
Contrast (mL), median (range)		180 (90–450)	150 (65–350)	0.041 ^†^
Per-lesion contrast (mL), median (range)		140 (45–300)	100 (29–250)	0.033 ^†^
Effective dose (mSv), median (range)		13 (6–43)	58 (14–203)	<0.001 ^†^
Per-lesion effective dose (mSv), median (range)		11 (3–43)	39 (14–126)	0.031 ^†^

CTCAE v5 = Common Terminology Criteria for Adverse Events version 5.0. * Chi-Square (χ^2^) test. ^†^ Unpaired *t*-test. ^‡^ Fisher’s exact test.

## Data Availability

The data presented in this study are available upon request from the corresponding author.

## Supplementary Materials

The following supporting information can be downloaded at: https://www.mdpi.com/article/10.3390/cancers16101925/s1, Figure S1: Flowcharts outlining the procedural steps of the HepACAGA technique and conventional ablation method; Figure S2: Overall survival separately presented for each tumor type.
