# Supplementary material for: Conventional versus Hepatic Arteriography and C-Arm CT-Guided Ablation of Liver Tumors (HepACAGA): A Comparative Analysis
Source: Cancers (Basel). 2024 May 18;16(10):1925. doi: 10.3390/cancers16101925 (PMC11119442; doi:10.3390/cancers16101925)
Supplement: Supplementary file 1 [file cancers-16-01925-s001.zip › cancers-2972606-supplementary.pdf]

Supplementary materials

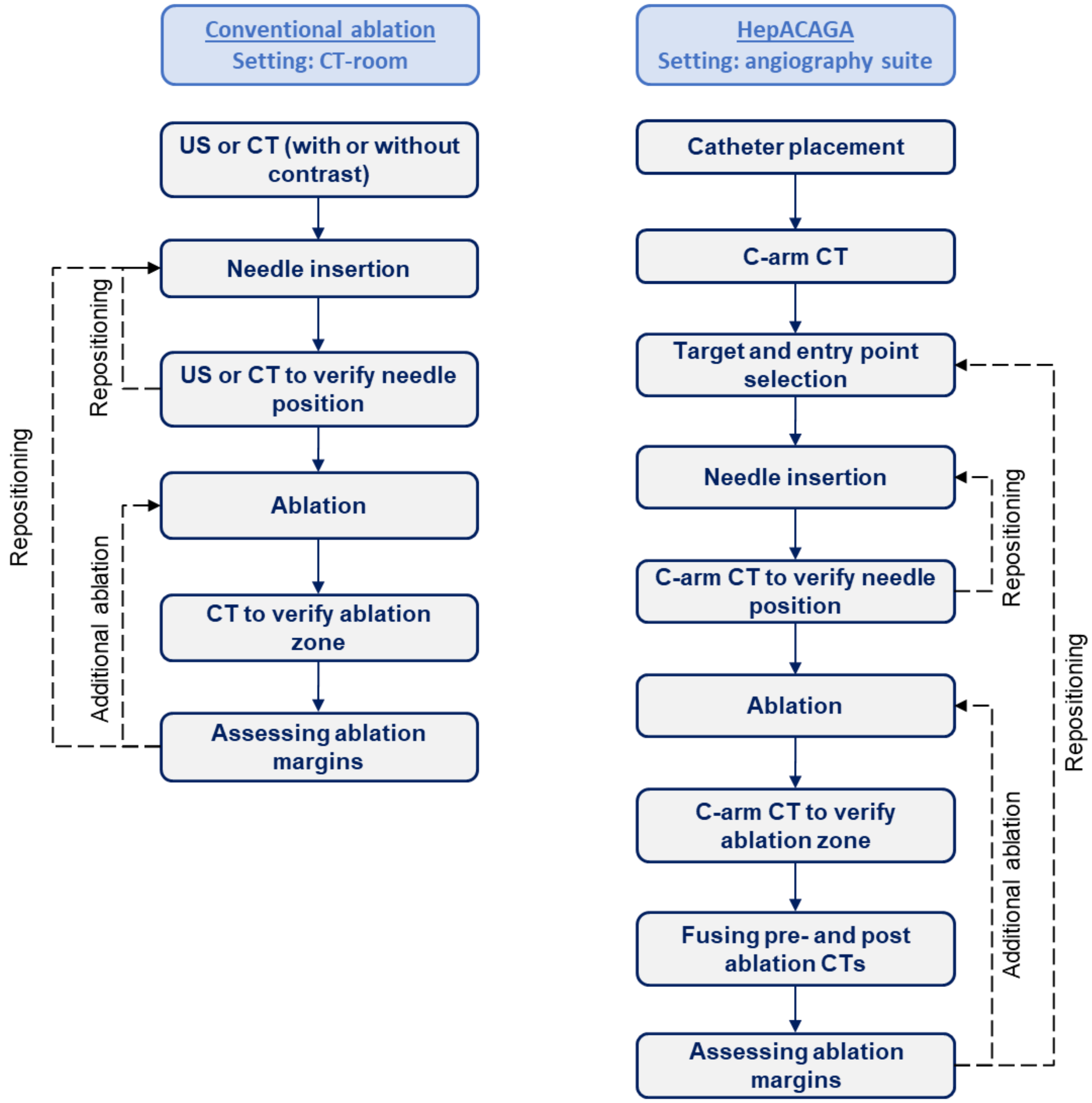

Figure S1. Flowchart of procedural steps involved in the conventional ablation method (left) and the HepACAGA technique (right).

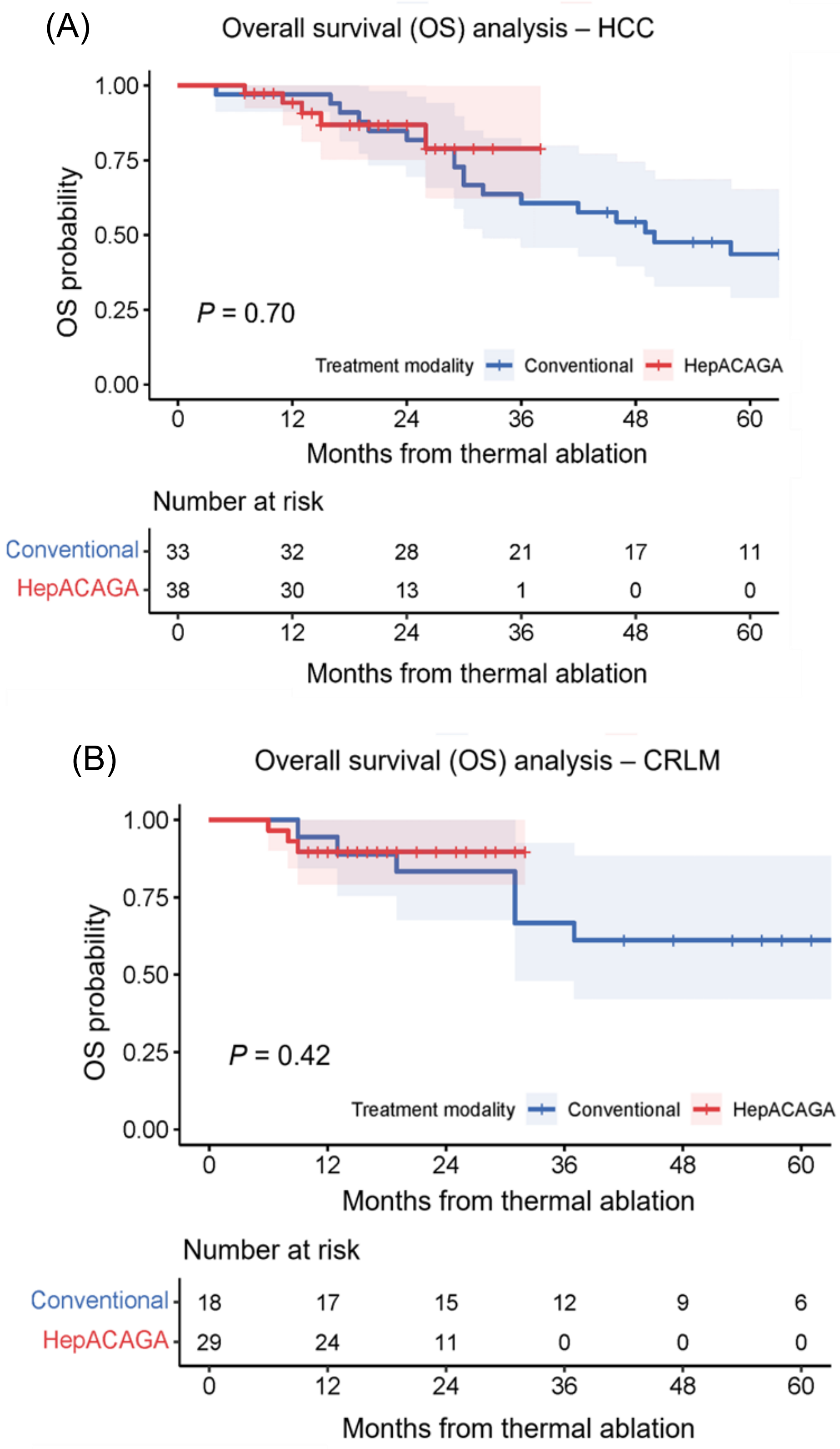

Figure S2. Kaplan–Meier survival curves illustrating the overall survival (OS) with 95% CI for both tumor types separately. Log-rank tests were used for comparison. The number at risk corresponds to either the number of HCC or CRLM present at each time point. (A) represents the HCC analysis; (B) demonstrates the CRLM analysis.
